# Supplementary material for: The interactome of intact mitochondria by cross-linking mass spectrometry provides evidence for coexisting respiratory supercomplexes
Source: Mol Cell Proteomics. 2017 Dec 8;17(2):216–32. doi: 10.1074/mcp.RA117.000470 (PMC5795388; doi:10.1074/mcp.RA117.000470)
Supplement: Supplemental Data [file supp_RA117.000470_133922_0_supp_23978_fzffwf.zip › spectra_annotation/mito_DR_spectra_annotation/449-1-2-1-1-1.pdf]

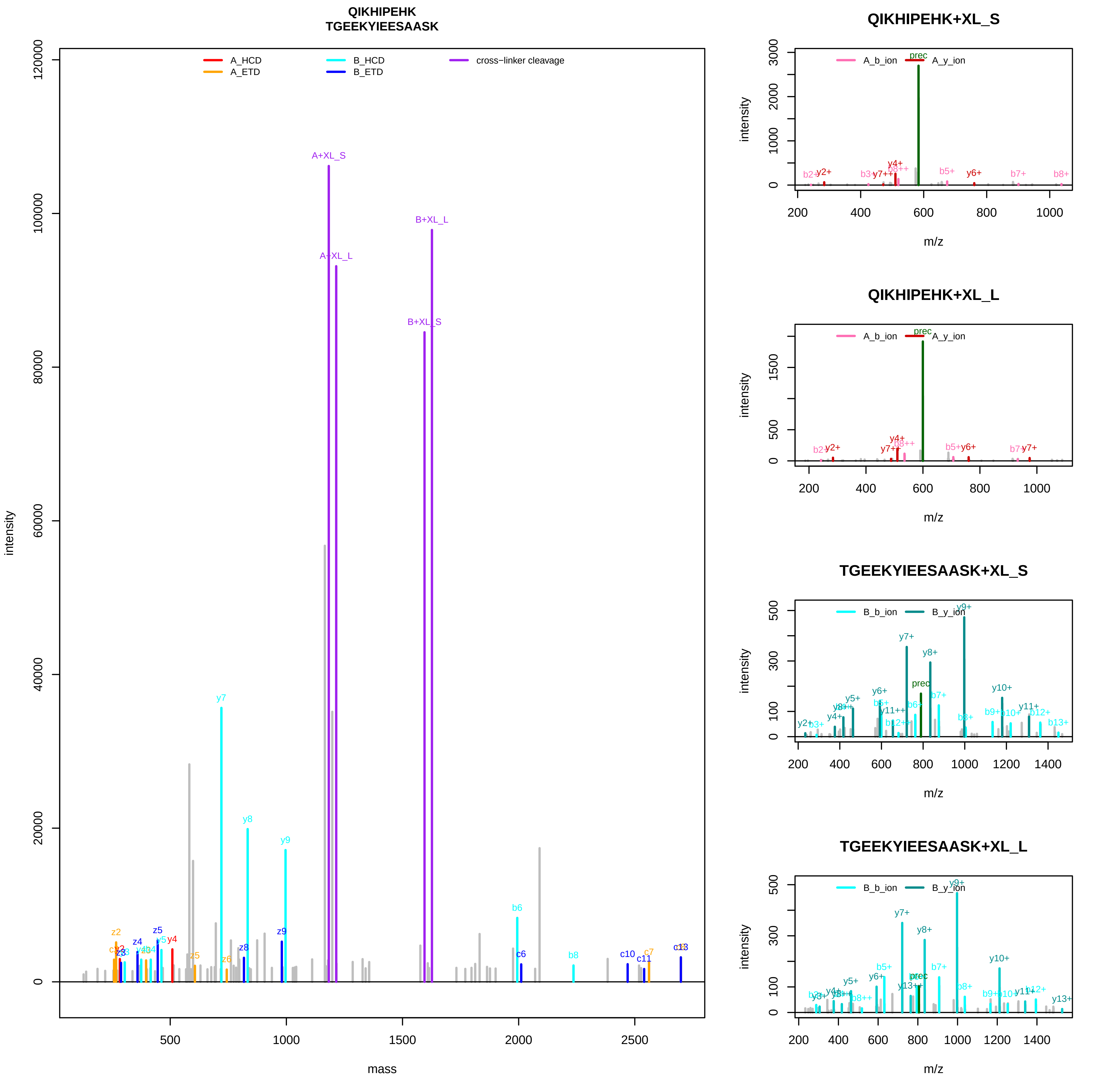

QIKHIPEHK
TGEEKYIEESAASK
A_HCD
A_ETD
B_HCD
B_ETD
cross−linker cleavage
A+XL_S
A+XL_L
B+XL_L
B+XL_S
y7
y8
y9
b6
b8
c6
c10
c11
c13
c7
z2
z4
z5
z8
z9
z5
z6
y4
y5
intensity
mass
0
20000
40000
60000
80000
100000
120000
500
1000
1500
2000
2500
QIKHIPEHK+XL_S
QIKHIPEHK+XL_L
TGEEKYIEESAASK+XL_S
TGEEKYIEESAASK+XL_L
A_b_ion
A_y_ion
B_b_ion
B_y_ion
prec
m/z
intensity
y2+
y4+
y6+
y7+
y7++
b2+
b3+
b5+
b7+
b8+
b8++
y9+
y10+
y11+
b13+
